# Shear Cap Size Selection Method Based on Parametric Analysis of ACI-318 Code and Eurocode 2 Standard

**DOI:** 10.3390/ma13214938

**Published:** 2020-11-03

**Authors:** Maciej Grabski, Andrzej Ambroziak

**Affiliations:** Faculty of Civil and Environmental Engineering, Gdansk University of Technology, 11/12 Gabriela Narutowicza Street, 80-233 Gdańsk, Poland; maciej.grabski.92@gmail.com

**Keywords:** reinforced concrete, slab-column connections, punching shear, shear cap, shear head

## Abstract

The scope of the paper is to propose a method for determining the size of shear caps in a slab–column-connections-reinforced concrete structure. Usually, shear heads are used to enhance slab–column connection, especially when the transverse reinforcement does not give the required punching shear load capacity. The dimensions of the shear head should provide the punching shear resistance of the connection inside and outside the enhanced region. The process of selecting the size of the shear head is iterative. The parametric analysis of the ACI 318 code and EC2 standard has the objective of indicating which control perimeter (inside or outside the shear head) has a decisive impact on the punching shear capacity of the connection. Based on the analysis, the authors propose methods for selecting the dimensions of the shear head with practical application examples. The paper is intended to provide scientists, civil engineers, and designers with guidelines to design the process of the slab–column connections with the shear caps.

## 1. Introduction

**Figure 1:**
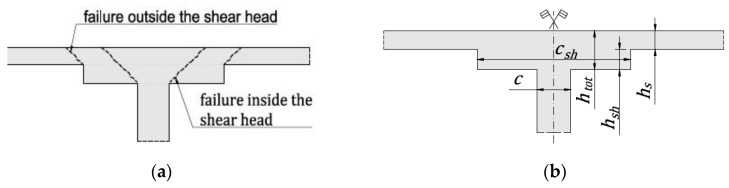
Slab–column connection with shear head: (**a**) potential failure region; (**b**) denotations: thickness of shear head ($h_{sh}$), thickness of slab ($h_{s}$), total thickness ($h_{tot}$), column span (*c*), shear head span ($c_{sh}$).

Flat reinforced concrete (RC) slab structures are used worldwide due to their many advantages over other types of structural solutions. The flat RC slabs give the possibility of free arrangement and use of the building area, and they are relatively simple and quick to build. One of the key issues in the design of slab–column structures is the support zone, which often requires enhancement, due to the accumulation of shear forces near the point support. The basic way to increase the shear capacity is to use punching shear reinforcement or boost slab thickness over the column (shear cap), like guidelines in the ACI 318 code [1] or in the EC2 standard [2]. The shear caps (popularly called shear heads) are commonly used, especially when the transverse reinforcement does not give the required load capacity. The second advantage of using the shear heads is the increased stiffness of the slab, which positively influences slab deformations. The design process of reinforced slab–column connections with shear head require verification of punching shear resistance conditions in the head zone and the slab zone outside the shear head; see Figure 1a. If the shear heads are used only to improve the punching capacity, the first condition determines the thickness of the shear head, while the second specified dimension of the shear-head. Designing the wrong thickness and/or size of the shear head may lead to failure of a slab-column connection and, in consequence, to reinforced concrete structure catastrophe.

Slab–column connections are a subject of much experimental research and theoretical investigation. Researchers have demonstrated the influence of many parameters on punching shear resistance, e.g., type and strength of concrete (see, e.g., [3,4,5,6]), support shape and dimension (see, e.g., [7,8,9,10]), quantity and distribution of longitudinal and transverse reinforcement (see, e.g., [11,12,13,14]), loading condition (see, e.g., [9,15,16]), membrane effect (see, e.g., [17,18,19]), scale effect (see, e.g., [11,20,21]), effect of the hole near to support (see, e.g., [22,23,24]), cyclic load (see, e.g., [25,26,27]). Investigations of these parameters have made it possible to develop methods and models to determine punching shear resistance. The physical model, which was well compatible with experimental results and explained the mechanism of failure due to punching shear, has been proposed by Kinnunen and Nylander [28]. Based on similar assumptions, the Critical Shear Crack Theory (CSCT) model was presented by Muttoni; see, e.g., [29,30,31]. Despite the developed physical methods, due to the complex nature of the issue and the multitude of factors influencing the load-bearing capacity of the connection between the plate and the column, most of the calculation methods were developed empirically. Taking into account the fact that the vast majority of the research concerns flat plates without the shear caps, it seems advisable to analyze the empirical methods in terms of punching through in a situation of shear cap enhancement.

It should be mentioned that the punching shear failure outside the shear head often deals with a large width $c_{sh}\gg 3d$. Compared to round support columns, the large square or rectangular columns generate a non-uniform distribution of the shear forces in the control perimeter. Additionally, the increase of the column dimension (regardless of the stress concentration in the corners) causes the nominal permissible tangential stress to decrease; see, e.g., [32,33]. These phenomena may cause the punching resistance not to be proportional to the length of the control perimeter. These effects have been taken into account in different ways over the years in the guidelines and standards. The current European standard does not take this fact into account, which has raised some concerns among researchers [9,33]. Some European countries have added restrictions on the punching for large supports in national annexes. This paper does not analyze this effect; it is adopted the basic EC2 standard provisions.

This research presents a unique parametric analysis of two calculation models of punching shear adopted in ACI-318 code [1] and EC2 standard [2]. The analysis is focused on determining the decisive control perimeter in slab–column connection with shear head on the punching shear resistance. This paper aims to propose the method for selecting the size of shear heads, which is based on the presented parametric analysis. Taking into account the fact that the process of selecting the size of shear head is iterative, this method can be useful in the design process of the slab–column connections with the shear caps. The practical application of the proposed method is confirmed by application examples (see Appendix A and Appendix B) estimating the minimum shear head dimension to obtain the required slab–column punching shear resistance. The influence of particular parameters on the selection of shear-head dimensions is also presented. The paper provides scientists, engineers, and designers with an analytical assessment of punching shear resistance of shear-head dimensions.

## 2. Analysis Method

The ratio of the head height ($h_{sh}$) to the slab height ($h_{s}$) and the ratio of head dimension ($c_{sh}$) to column dimension (c, see Figure 1b, column span (*c*)) are assumed as main parameters and defined as follows:

(1) $$\begin{array}{cc} \alpha_{1}=\frac{h_{sh}}{h_{s}}\;, & \alpha_{2}=\frac{c_{sh}}{c}\; \end{array}$$

At this point, a requirement should be specified for which values of the main parameters the decisive condition for the punching shear resistance of slab–column connection becomes the criterion inside the shear heads. The condition that determines the above requirement is that the rate of intensity (defined as the ratio of punching force ($V_{E,sh}$, $V_{E,s}$) to punching resistance ($V_{R,sh}$, $V_{R,s}$)) in the control perimeter inside the shear head is greater than the rate of intensity in the control perimeter outside the head:

(2) $$\frac{V_{E,sh}}{V_{R,sh}}/\frac{V_{E,s}}{V_{R,s}}=\omega_{sh}/\omega_{s}\;\geq 1.0$$

The parameters $\omega_{sh}$ and $\omega_{s}$ define the punching shear rate of intensity in the control perimeter inside the head and outside the head, respectively.

The value of parameters $\omega_{i}$ (index ‘*i*’ means the control perimeter under consideration) may be determined for EC2 standard [2], as follows:

(3) $$\omega_{i}=\frac{\beta_{i}\cdot V_{E,i}}{\left(0.18\cdot k_{i}\cdot {\left(100\cdot \rho_{l,i}\cdot f_{ck}\right)}^{\frac{1}{3}}\right)\cdot u_{i}\cdot d_{i}}$$

where β is coefficient and represents the effect of an unbalanced moment, $\rho_{l}$ is the reinforcement ratio for longitudinal reinforcement, $f_{ck}$ is characteristic compressive cylinder strength of concrete at 28 days in MPa, coefficient $k=1+\sqrt{200/d}\leq 2$, u is the length of the control perimeter, d is the effective slab height.

The value of parameters $\omega_{i}$ for ACI-318 code [1] can be specified as:

(4) $$\omega_{i}=\frac{\gamma_{i}\cdot V_{E,i}}{v_{c}\cdot b_{0}\cdot d_{i}}$$

where γ represents the influence of the unbalanced bending moment acting on the joint, $v_{c}$ is the nominal shear strength, and $b_{0}$ is the length of the control perimeter.

The value of β (and γ) is greater the smaller the distance from the column face to the control perimeter under consideration. Therefore, the ratio $\beta_{sh}/\beta_{s}$ (and $\gamma_{sh}/\gamma_{s}$) will always be greater than 1, and the basic analysis condition (Equation (2)) be satisfied, regardless of the β (or γ) value. Thus, in the present analysis, the value of β (and γ) is equal to 1.

**Figure 2:**
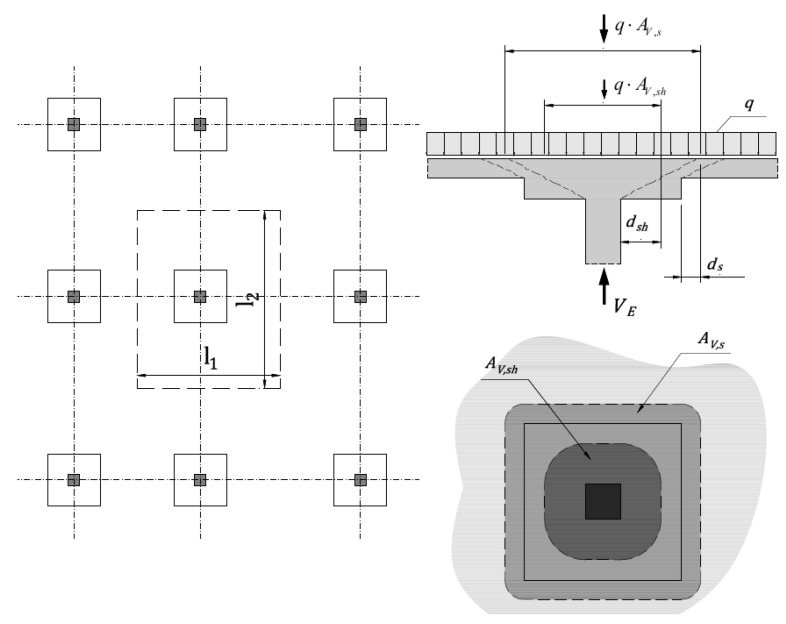
The shear force acting on the control perimeter under consideration.

**Figure 3:**
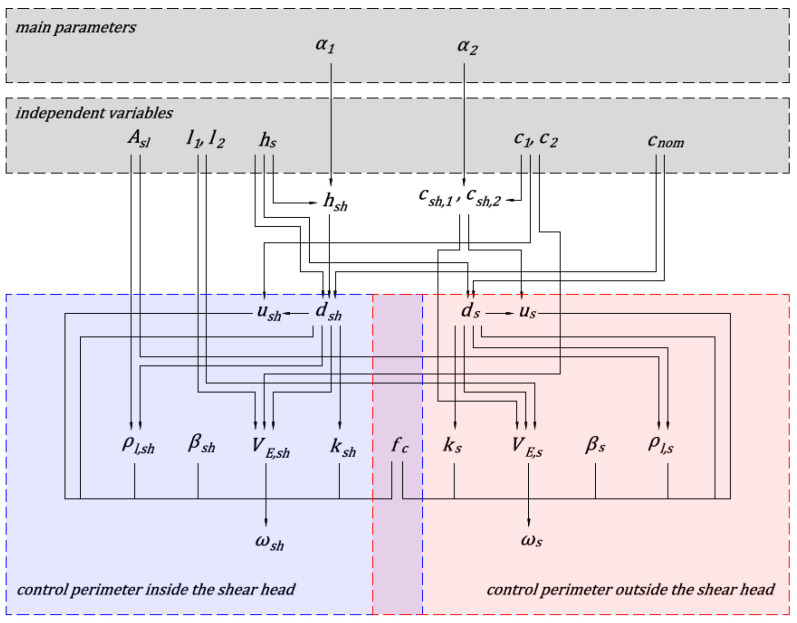
Parameters dependency diagram for the EC2 standard.

**Figure 4:**
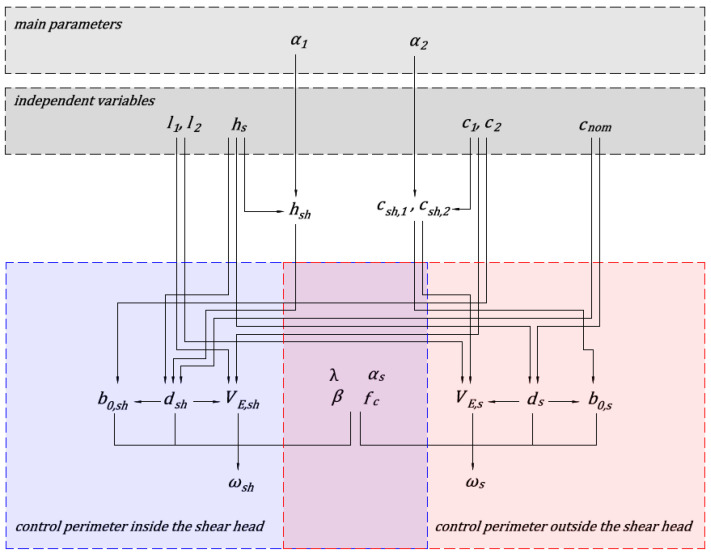
Parameters dependency diagram for ACI 318 code.

Assuming a symmetrical slab–column connection (square head and column, see Figure 2) under uniformly distributed loads, the punching force acting on the control perimeter under consideration can be calculated from the equations:

(5) $$\begin{array}{l} V_{E,sh}=V_{E}-\Delta V_{sh}=q\cdot A_{V}-q\cdot A_{V,sh}\\ V_{E,s}=V_{E}-\Delta V_{s}=q\cdot A_{V}-q\cdot A_{V,s} \end{array}$$

where $A_{V}=l_{1}\cdot l_{2}$ [m^2^] is an area of uniformly distributed load, *q* [kPa] is the magnitude of the loads. The area $A_{V,s}$ is assumed to be equal to the size of the shear head increased by $d_{s}$, and area $A_{V,sh}$ is assumed to be equal to the size of the column increased by $d_{sh}$. The concrete class and longitudinal reinforcement cross-section (*A*_sl_) and cover of the longitudinal reinforcement (*c*_nom_) are assumed to be identical in the slab and the shear head. In the calculations, the membrane forces in the floor slab are not taken into account. No shear reinforcement is assumed. Based on the described assumptions, the independent variables taken into account in the parametric analysis are determined. Figure 3 and Figure 4 show diagrams of the dependence of parameters of the analyzed methods. The value of the $\alpha_{1}$ parameter depends on the assumptions of the analyzed national standard and calculation case under consideration. The national standards provide methods for determining the total height of shear cap ($h_{tot}=h_{s}+h_{sh}$). Depending on the calculation case, the total height of the head is selected to fulfil capacity conditions. Based on the shear head height and slab thickness parameter $\alpha_{1}$ is calculated. The $\alpha_{2}$ parameter is responsible for specifying the size of shear head, and is determined based on the proposed method for selecting the size of shear caps based on the parametric analysis.

## 3. Results

**Figure 5:**
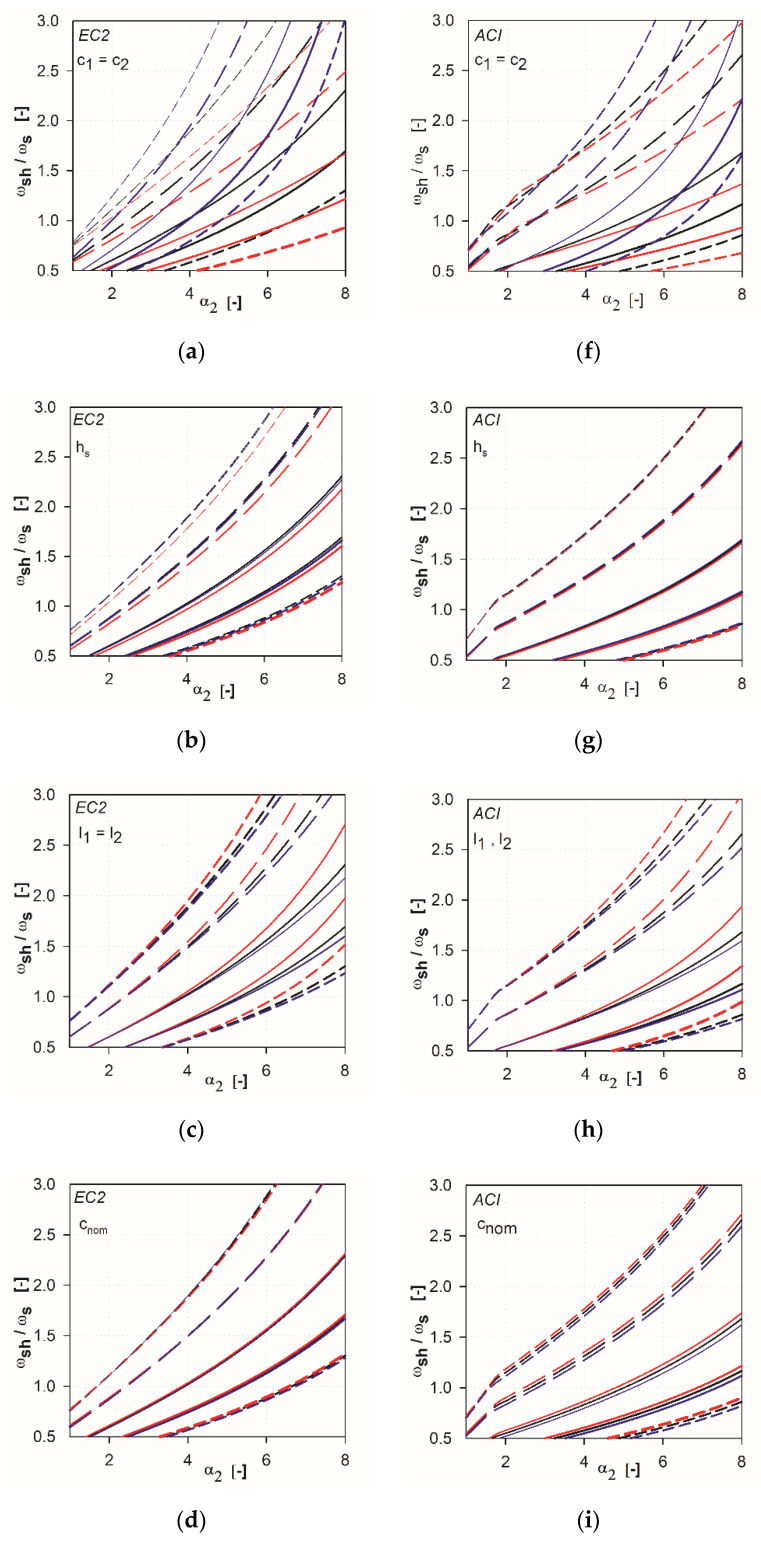

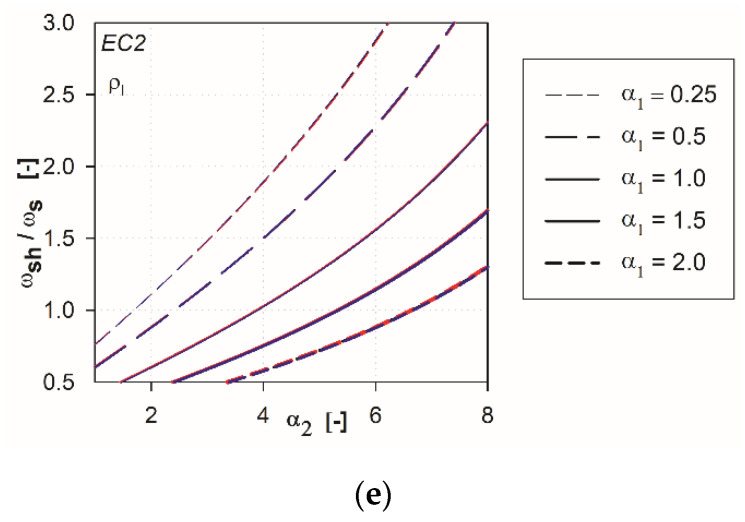
Independent variables impact on the results of the analysis: (**a**) and (**f**) column dimension; (**b**) and (**g**) slab height; (**c**) and (**h**) slab span; (**d**) and (**i**) reinforcement cover; (**e**) slab reinforcement ratio.

The analysis is based on deriving the relationships between $\omega_{sh}/\omega_{s}$ and the parameter $\alpha_{2}$ for different values of $\alpha_{1}.$ Each of the relationships for a particular set of independent variables was obtained by calculating the values of $\omega_{sh}/\omega_{s}$ (Formulas (3) or (4)) for the assumed parameter $\alpha_{1}$ by changing parameter $\alpha_{2}$ every 0.25, starting from 1 to 10. These relationships are obtained for different values of independent variables. For each independent variable, three values are considered: lower value, basic value, upper value (see Table 1). The values are selected according to the guidelines used in practice. In the first step of the analysis, we calculated the dependencies by examining each of the independent variables separately. Considering a particular variable, the value of this one independent variable changed, while the other variables remained constant (as basic values). The results of the calculations are presented in Figure 5a–e for EC2 standard and in Figure 5f–i for ACI 318 code. The lower value of the analyzed variable is shown in red, the basic value in black, and the upper value in blue.

In the case of EC2 standard [2], it can be shown that in the range $\omega_{sh}/\omega_{s}\;<1$, the reinforcement ratio for longitudinal reinforcement and the concrete cover does not affect the results obtained. The minimal influence is visible for the slenderness of the slab and the slab height. The greatest variability is characterized by column size (see Figure 5a,f), because the size of the support directly affects the parameter $\alpha_{2}$. In the case of ACI318 code [1], the most changeable is the graph with the column size parameter under consideration. The minimum variation in results is visible for the slab slenderness parameter and the upper reinforcement cover.

**Figure 6:**
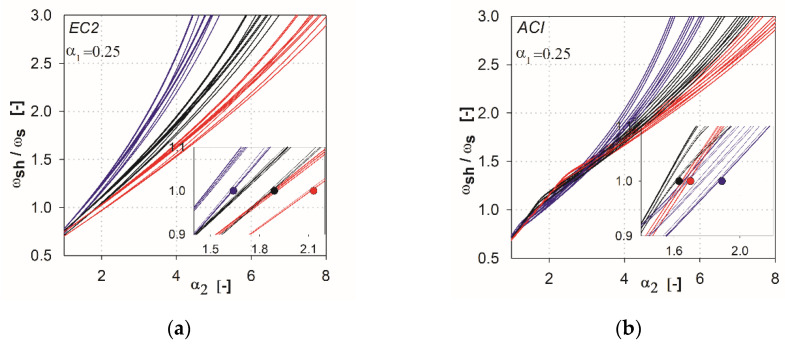
Graphs obtained for the parameter $\alpha_{1}=0.25$: (**a**) for the EC2 standard; (**b**) for the ACI code.

**Figure 7:**
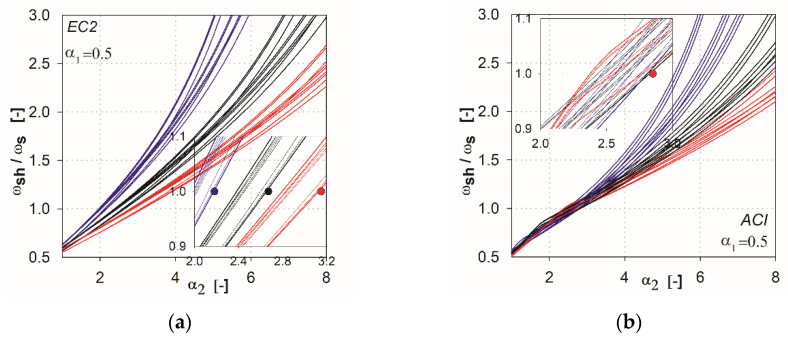
Graphs obtained for the parameter $\alpha_{1}=0.5$: (**a**) for the EC2 standard; (**b**) for the ACI code.

**Figure 8:**
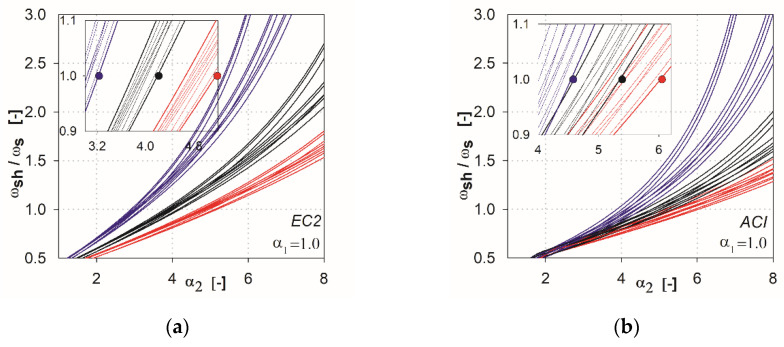
Graphs obtained for the parameter $\alpha_{1}=1.0$: (**a**) for the EC2 standard; (**b**) for the ACI code.

**Figure 9:**
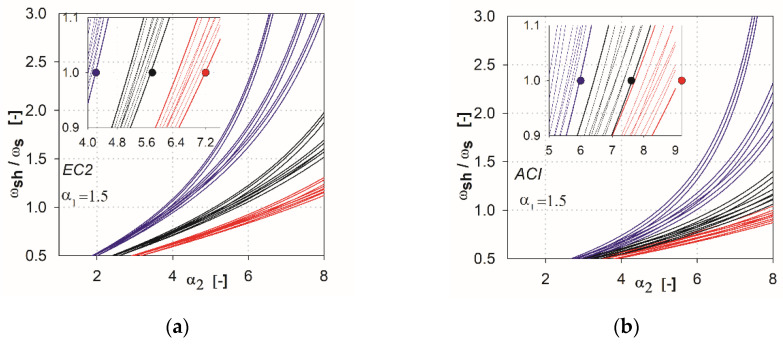
Graphs obtained for the parameter $\alpha_{1}=1.5$: (**a**) for the EC2 standard; (**b**) for the ACI code.

**Figure 10:**
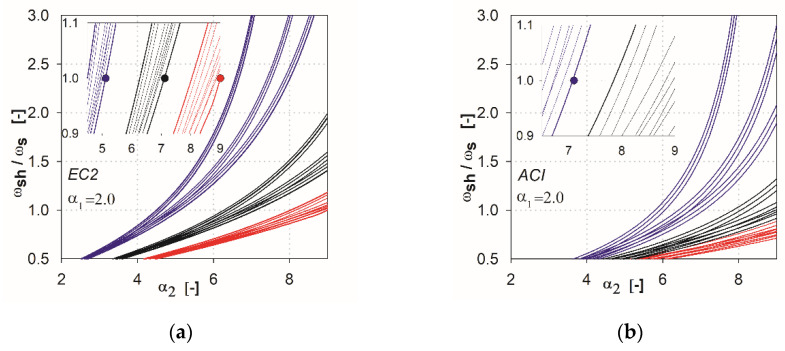
Graphs obtained for the parameter $\alpha_{1}=2.0$: (**a**) for the EC2 standard; (**b**) for the ACI code.

In the next step of the analysis, we made additional calculations by combining the parameters that show the variability of results. For the EC2 standard [2], the combinations are created from the parameters *l* (*l*_1_, *l*_2_), *h*_s_, *c* (*c*_1_, *c*_2_), whereas for ACI, they are *l* (*l*_1_, *l*_2_), *c*_nom_, *c* (*c*_1_, *c*_2_). This resulted in 27 combinations of the independent variables for each method (three independent variables with three values each). In Figure 6, Figure 7, Figure 8, Figure 9 and Figure 10, the comparisons for different values of the parameter $\alpha_{1}$ are given. We noted that the calculated curves are grouped into three categories, depending on the column size parameter. In the figures, these groups were marked by colors (red for the parameter *c*_1_ = *c*_2_ = 1.5 *h*_s_, black for *c*_1_ = *c*_2_ = 2.0 *h*_s_, blue for *c*_1_ = *c*_2_ = 3.0 *h*_s_). Based on these data, it can be shown for which values of the main parameters Equation (2) is satisfied. The maximum value of the parameter $\alpha_{2}$, for which the control section strength inside the shear head is equal to the control section strength outside the head, is determined by $\left(\frac{\omega_{sh}}{\omega_{s}}\left(\alpha_{2}\right)=1\right)$. The values are presented in the form of points on the Figure 6, Figure 7, Figure 8, Figure 9 and Figure 10, and are collected in Table 2 and Table 3. These points mean that, for assumed values of the parameter $\alpha_{1}$ and column size $c_{1}=c_{2}$, a minimum parameter $\alpha_{2}$ should be used to satisfy the condition defined in Equation (2).

**Figure 11:**
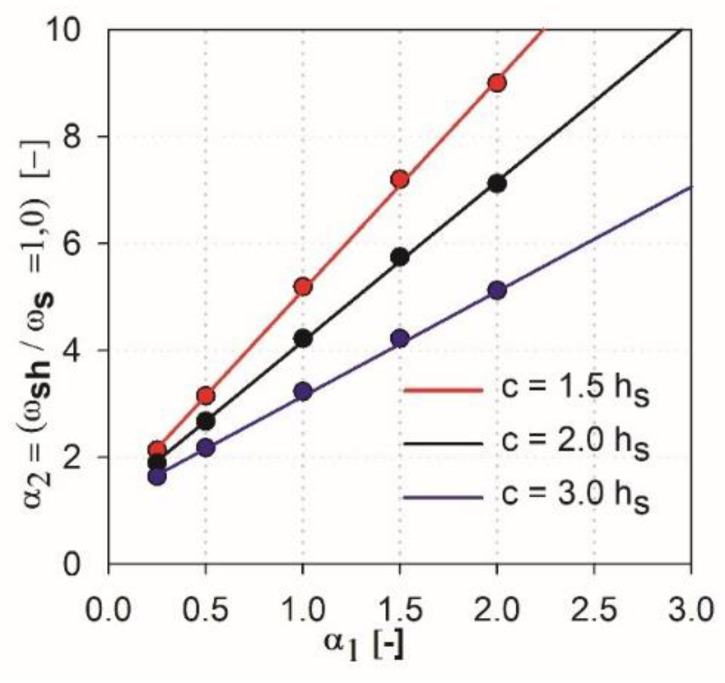
Summary of the analysis results—EC2.

The approximation functions for the performed EC2 standard analysis (see Figure 11) can be determined, as:

(6) $$\alpha_{2}=3.95\cdot \alpha_{1}+1.18,\;\mathrm{for}\;c_{1}=c_{2}=1.5\;h_{s}$$

(7) $$\alpha_{2}=3.0\cdot \alpha_{1}+1.18,\;\mathrm{for}\;c_{1}=c_{2}=2.0\;h_{s}$$

(8) $$\alpha_{2}=2.0\cdot \alpha_{1}+1.18,\;\mathrm{for}\;c_{1}=c_{2}=3.0\;h_{s}$$

The function $\alpha_{2}\;\left(c,\alpha_{1}\right)$, which approximates all the results, the relationship of the linear function directional coefficient depending on the value of $c/h_{s}$ (where $c=c_{1}=c_{2}$) should be determined. Such a function is proposed as a parabolic relationship (this assumption allows to avoid additional conditions for particular ranges in the case when the lower order of approximation function would be used, e.g., the piecewise linear approximation function) and takes the form

(9) $$a\left(\frac{c}{h_{s}}\right)=0.58\cdot {\left(\frac{c}{h_{s}}\right)}^{2}-3.93\cdot \left(\frac{c}{h_{s}}\right)+8.53$$

Finally, the functions $\alpha_{2}\;\left(c,h_{s},\alpha_{1}\right)$, which approximate obtained results of parametric analysis, are derived for the EC2 standard as

(10) $$\alpha_{2}=\left(0.58\cdot {\left(\frac{c}{h_{s}}\right)}^{2}-3.93\cdot \left(\frac{c}{h_{s}}\right)+8.53\right)\cdot \alpha_{1}+1.18$$

**Figure 12:**
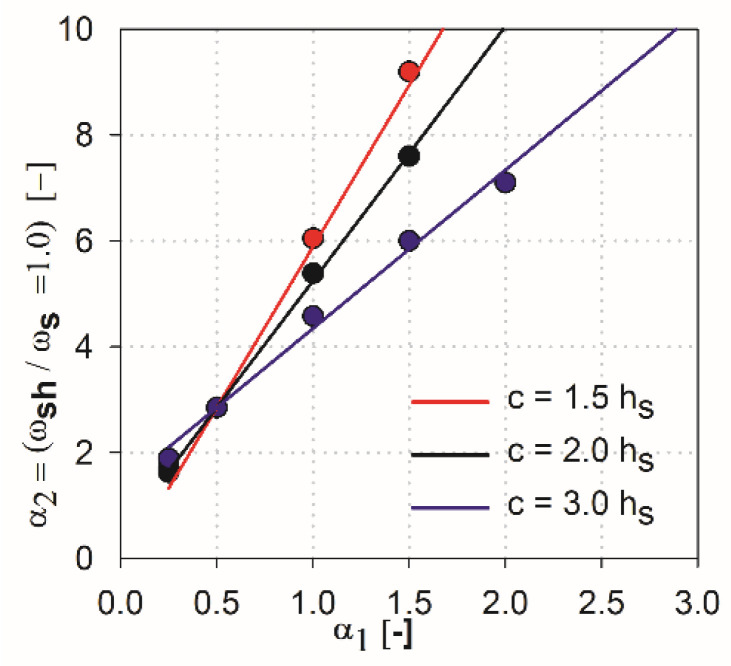
Summary of the analysis results—ACI 318.

Following, by performing operations like above for the ACI318 code results of the analysis (see Figure 12) and take into account that the value of parameter $\alpha_{2}$ for $\alpha_{1}=0.5$ is 2.85 (see Table 3, ACI318), the approximating function for ACI318 code is derived as:

(11) $$\alpha_{2}=\left(0.51\cdot {\left(\frac{c}{h_{s}}\right)}^{2}-4.34\cdot \left(\frac{c}{h_{s}}\right)+11.46\right)\cdot \left(\alpha_{1}-0.5\right)+2.85$$

## 4. Discussion

It can be stated that the higher the shear cap (higher parameter $\alpha_{1}$), the greater influence that independent variables of particular methods have on this analysis, and the greater the difference in the results that occur. In the case of the ACI318 code [1] for parameters $\alpha_{1}=0.25$ and $\alpha_{1}=0.5$ within $\omega_{sh}/\omega_{s}\;=1$, relatively convergent results can be observed. The values of independent variables have a relatively low impact on the obtained results. Three groups of functions can be identified, depending on the analyzed support dimension only for parameters $\alpha_{1}$ higher than 0.5. For the EC2 standard [2], from the initial values of the parameter $\alpha_{1}$ the function groups related to the assumed column dimension are clearly separated. For high thicknesses of shear heads ($\alpha_{1}=1.5\;and\;\alpha_{1}=2)$, the values $\alpha_{2}$ at which the curves reach $\omega_{sh}/\omega_{s}\;=1$ are very large, especially for the ACI318 code; see Figure 12. For example, for the parameter $\alpha_{1}=1.5$ with the column dimension equal to $3.0\;h_{s}$ (blue color curves, see Figure 12), the value $\omega_{sh}/\omega_{s}\;=1$ is reached at $\alpha_{2}=\sim 6.0$ (for the EC2 standard, it would be $\alpha_{2}=\sim 4.2$). In this case, in order for the load capacity of the control perimeter outside the head to exceed the load capacity of the perimeter inside the shear head, the thickness should be assumed to be about $\sim 6.0\cdot 3\;h_{s}=18\;h_{s}$. Considering the typical slenderness of the slab ($l_{1}=33\;h_{s}$), the thicker part would occupy more than half of the floor area. Such design situations are not common in practice (except for heads used due to ceiling deflection—drop panels). Therefore, it can be assumed that, for heads with the parameter $\alpha_{1}\geq 1.5$, the dimension (width) of the shear head should be selected by checking the resistance condition in the cross-section outside the head iteratively. For very small thicknesses ($\alpha_{1}<0.25$), the approximation functions do not give the desired results, because in values $\alpha_{1}=0$, they should converge to $\alpha_{2}=1$. However, this is irrelevant, given the fact that such low heads do not exist in practice. It should be assumed that the approximation functions give correct and useful results for the values of $0.25<\alpha_{1}<1.5$.

The authors propose the shear caps size dimensions selection method based on the parametric analysis. In the EC2 standard [2] case, the algorithm for the proposed method can be specified as follows:

- Determine the required total thickness of shear head ($h_{tot}$) from the design data (geometry, loads, material, etc.). During the above calculations, the condition set in the assumptions must be verified: $v_{Rd,c}\geq v_{min}$.
- Calculate parameter $\alpha_{1}$ and $c_{s}/h_{s}$.
- Compute the parameter $\alpha_{2}$ (see Equation (10)) and the shear head dimensions, as: $c_{sh,1}=\alpha_{2}\cdot c_{s,1}$ and $c_{sh,2}=\alpha_{2}\cdot c_{s,2}$.

It should be noted that the proposed method can be applied to rectangular columns. In this case, $c_{s}=\min\left(\;c_{s,1}\;;c_{s,2\;}\right)$ should be used in the parameter $c_{s}/h_{s}$, and the head dimension should be determined as in the given algorithm (rectangular shear head with the same proportion of sides as the column dimensions is specified). The assumption of parameter $c_{s}=\min\left(\;c_{s,1}\;;c_{s,2\;}\right)$ causes results on the safe range.

On the other hand, for the ACI318 code [1], the algorithm can be described as:

- Determine the required total thickness of shear head ($h_{tot}$) from the design data (geometry, loads, material, etc.).
- Compute the parameters $\alpha_{1}$ and $c_{s}/h_{s}$.
- Specify the parameter $\alpha_{2}$ (see Equation (11)) and the shear head dimensions: $c_{sh,1}=\alpha_{2}\cdot c_{s,1}$ and $c_{sh,2}=\alpha_{2}\cdot c_{s,2}$.

In the case of rectangular support, the value $c_{s}$ should be determined according to the following conditions: while $0.25\leq \alpha_{1}<0.5$, the $c_{s}=\max\left(\;c_{s,1}\;;c_{s,2\;}\right)$, and for $\alpha_{1}\geq 0.5\to c_{s}=\min\left(\;c_{s,1}\;;c_{s,2\;}\right)$.

**Figure A1:**
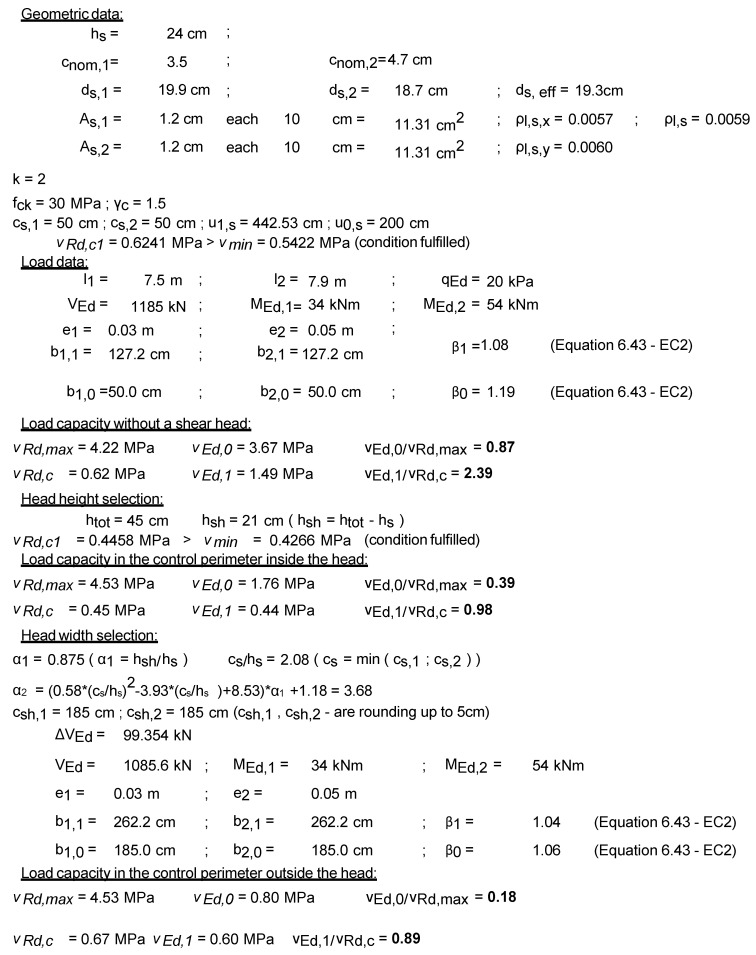
Verification example no. 1—EC2.

**Figure A2:**
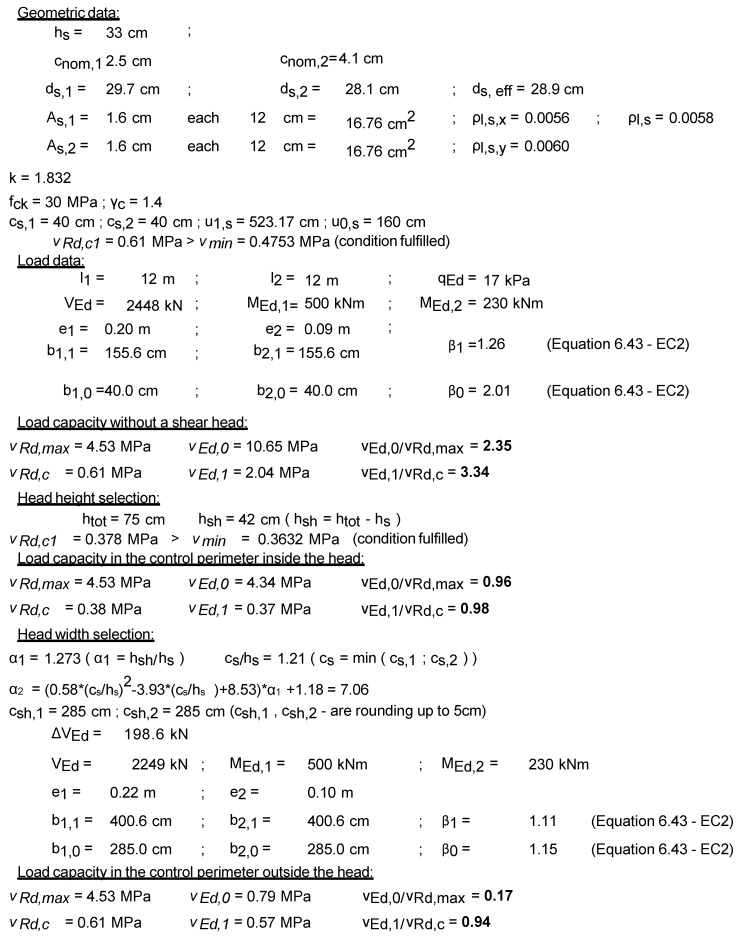
Verification example no. 2—EC2.

**Figure A3:**
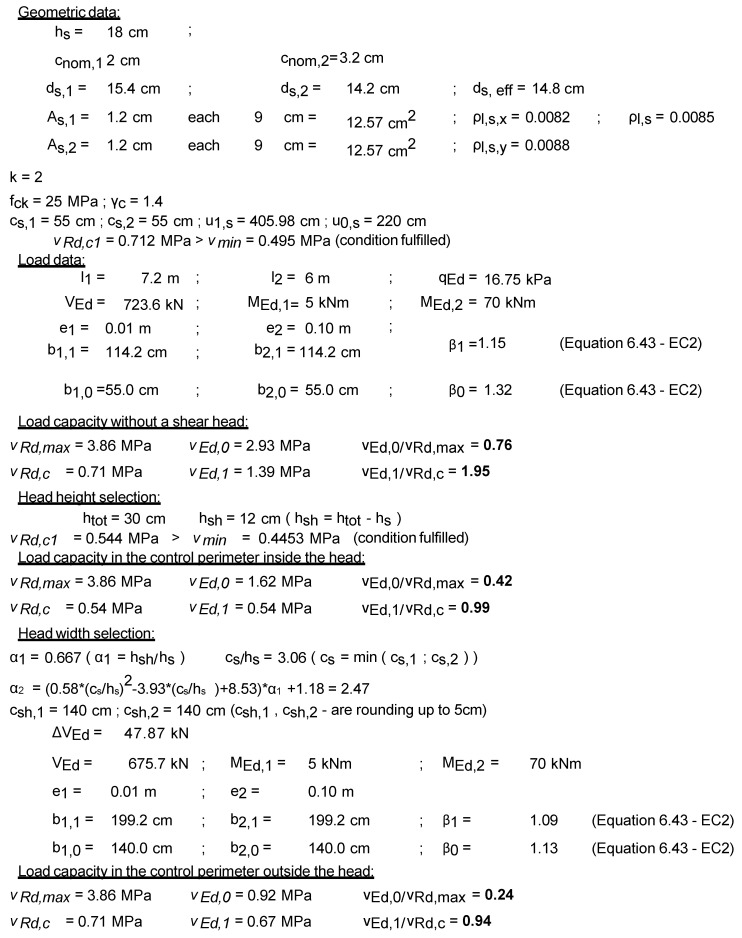
Verification example no. 3—EC2.

**Figure A4:**
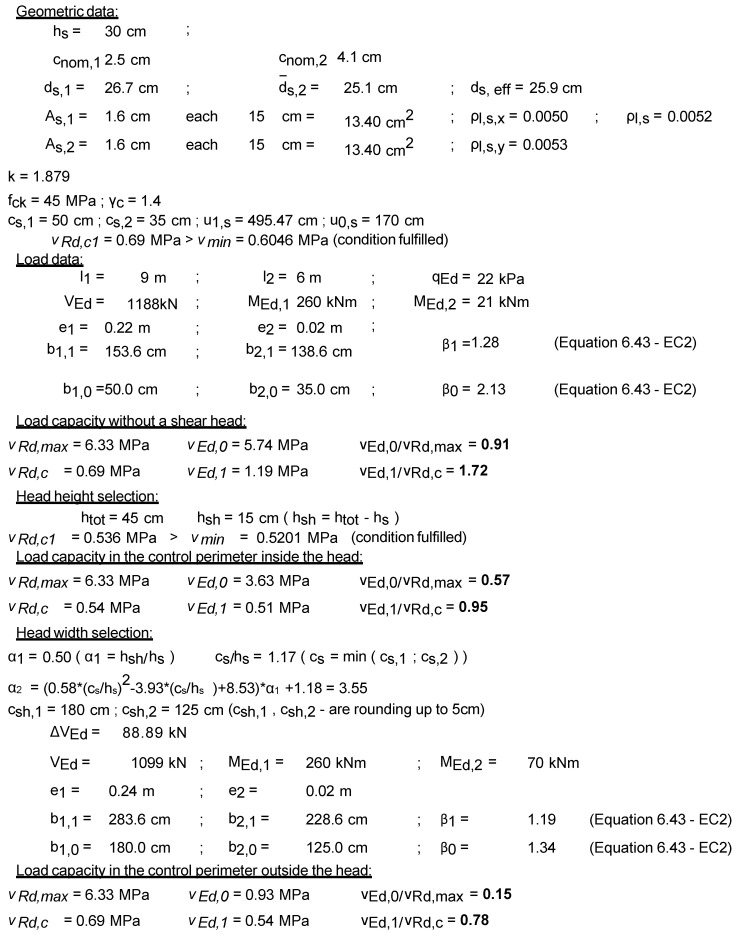
Verification example no. 4—EC2.

**Figure A5:**
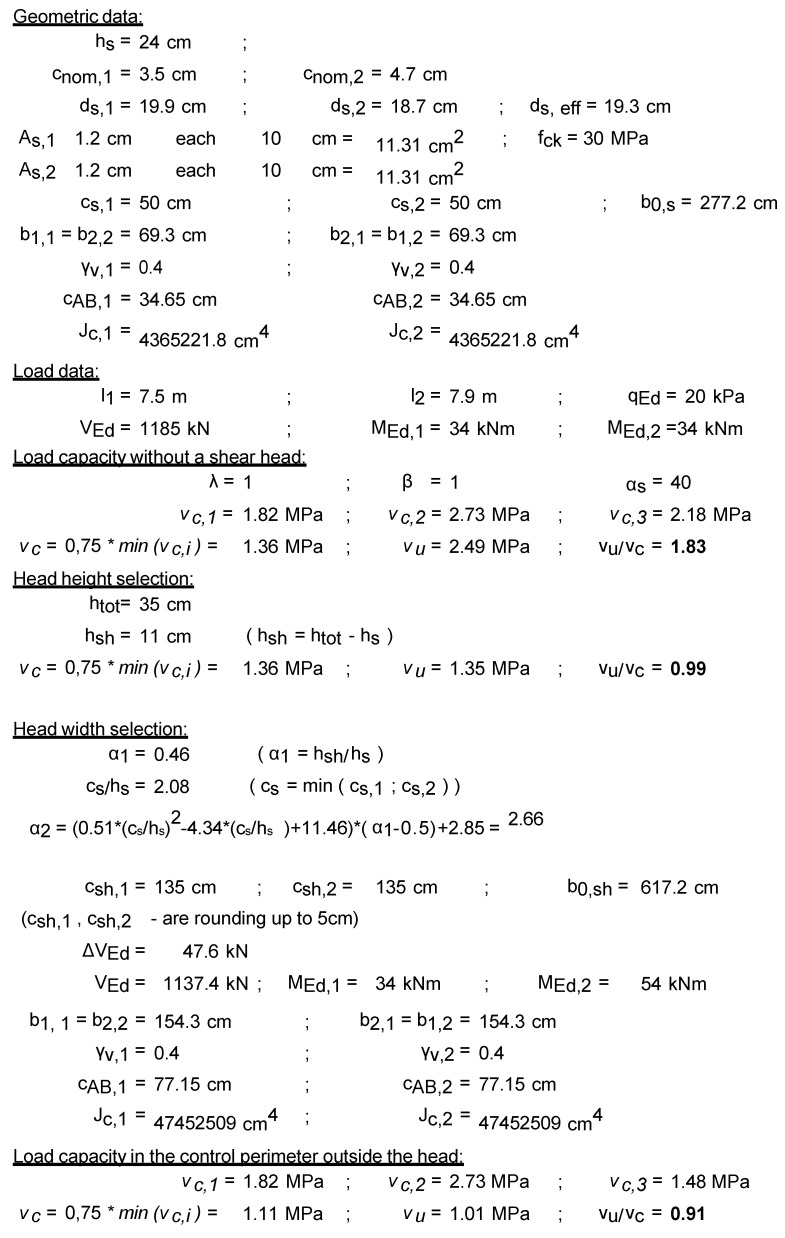
Verification example no. 1—ACI 318.

**Figure A6:**
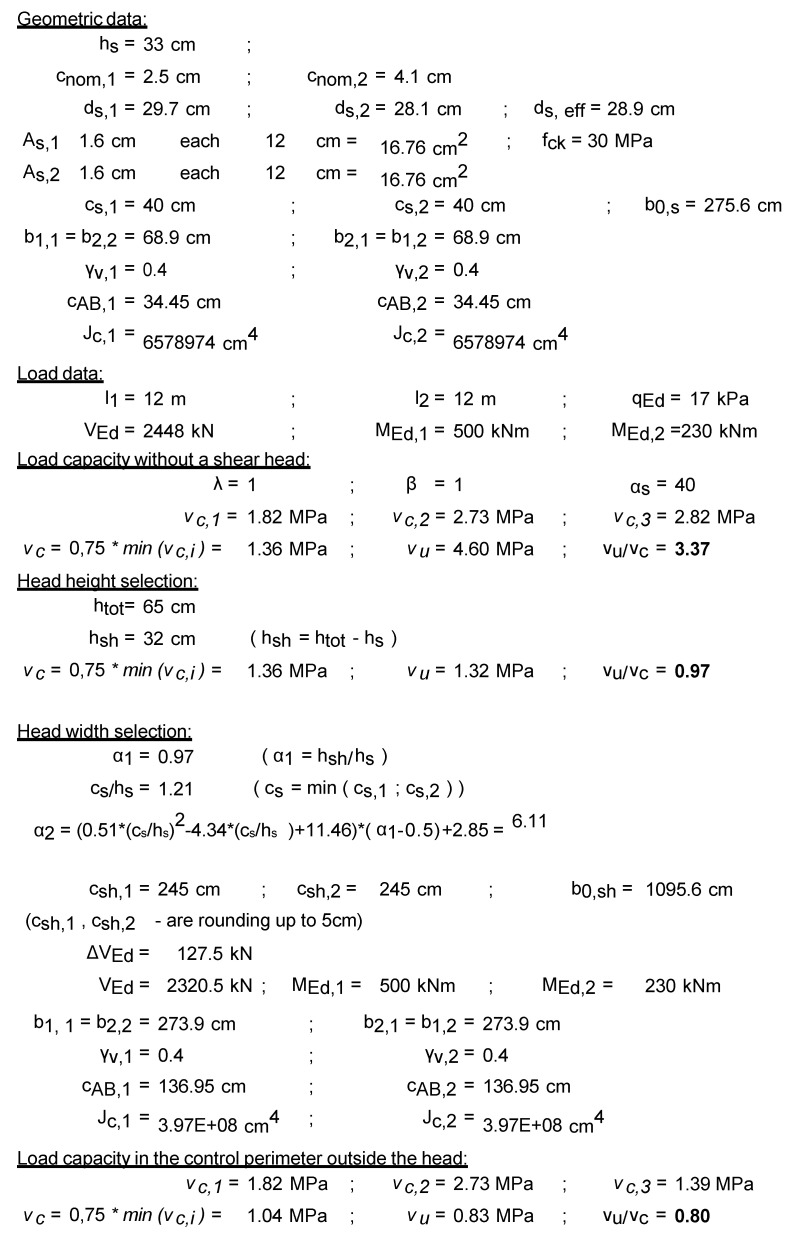
Verification example no. 2—ACI 318.

**Figure A7:**
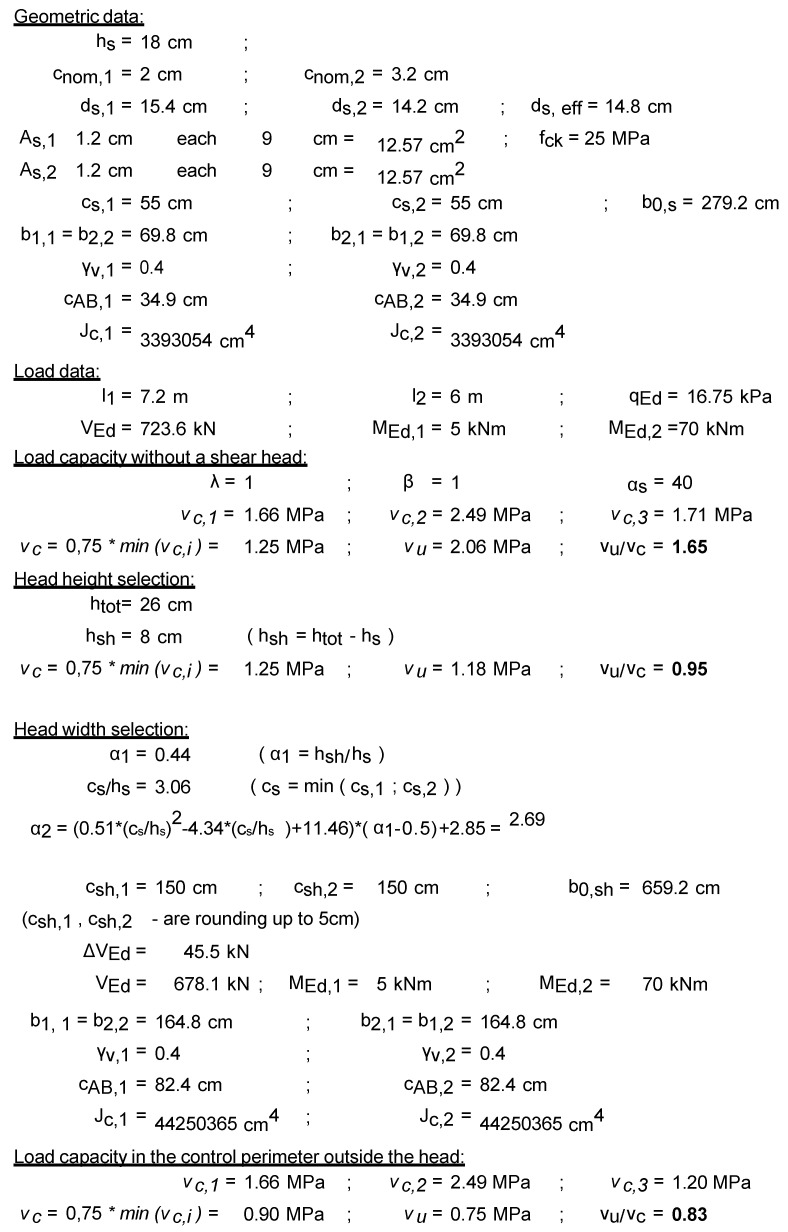
Verification example no. 3—ACI 318.

**Figure A8:**
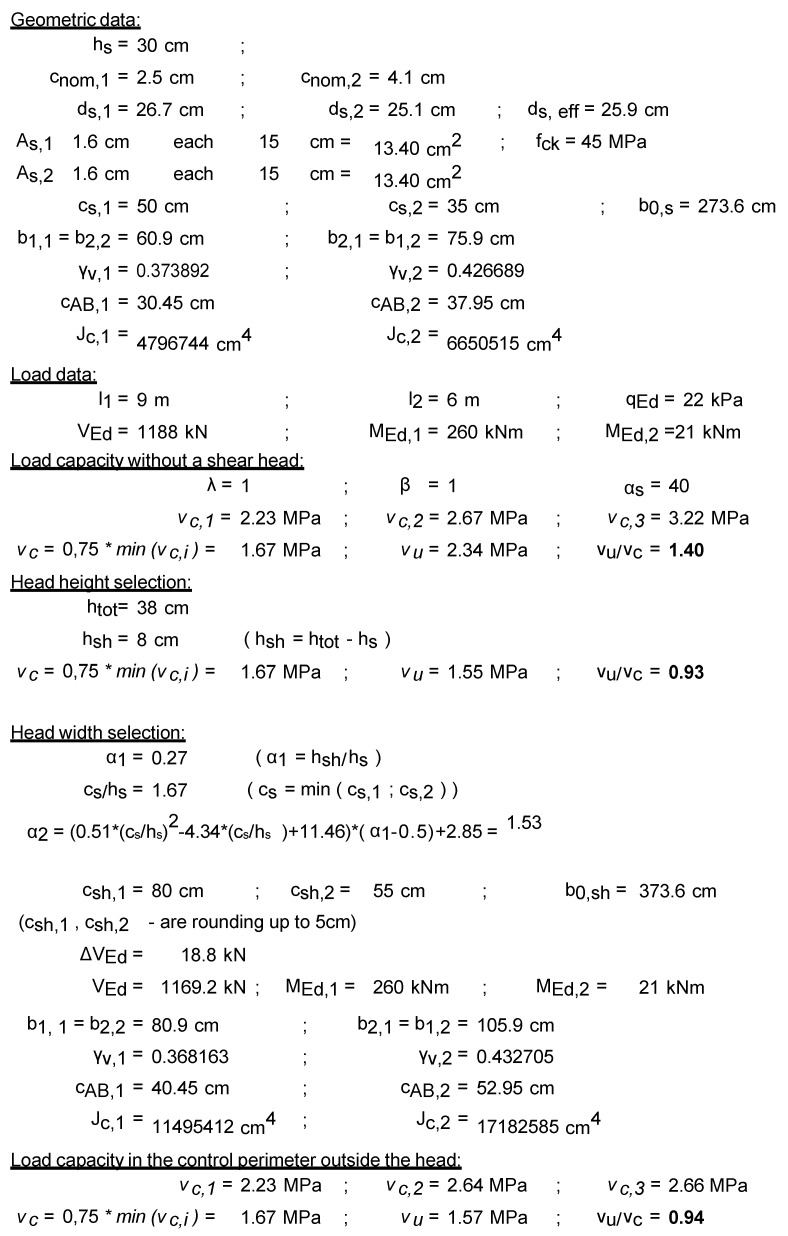
Verification example no. 4—ACI 318.

Appendix A and Appendix B present the verification examples (see Figure A1, Figure A2, Figure A3, Figure A4, Figure A5, Figure A6, Figure A7 and Figure A8) for the shear heads selecting size, according to the proposed method. The notations used in Figure A1, Figure A2, Figure A3 and Figure A4 and Figure A5, Figure A6, Figure A7 and Figure A8 are collected and described in Table A1 and Table A2, respectively. The slab–column structure (see Figure 2) under uniformly distributed loads is taken under consideration in the calculations. The different slab spans, design values of total distributed load, slab thickness, and column dimension are taken into account in the calculations. In the dimensioning examples, the geometric data of slab–column structure and the loadings data are firstly accepted. Next, the punching shear resistance of reinforced concrete slab is determined. The thickness of the slab is insufficient for carrying shear loadings, and the shear head height is specified. Next, the shear load capacity in the control perimeter inside the head is calculated. The shear head width according to proposed equations is defined. Finally, the shear load capacity in the control perimeter outside the shear head is verified.

To fulfil standards requirements, the proper shear head thickness and dimensions are specified. Table 4 summarizes the accepted design shear head dimensions for each verification example (see Figure A1, Figure A2, Figure A3, Figure A4, Figure A5, Figure A6, Figure A7 and Figure A8). It should be pointed out that the punching shear resistance without transverse reinforcement is higher, according to the ACI318 code [1], like in the EC2 standard [2] (see, e.g., [34]). Using the guidelines of the ACI318 code, a significantly smaller thickness of the shear head is determined; see Table 4.

## 5. Conclusions

The parametric analysis according to design guidelines given in the EC2 standard [2] and the ACI318 code [1] is carried out. The authors determined the decisive control perimeter (inside or outside the shear head) in slab–column connection with shear head on the punching shear resistance. The specified function (see Equations (10) and (11)) allows for specifying the necessary dimension of the shear head in the slab–column connection. The method for selecting the shear head dimension is proposed. The verification examples (see Appendix A and Appendix B) show the practical application of the proposed methods of estimating the minimum shear head dimension to obtain the required slab–column punching shear resistance. The method is designed to give safe results under all boundary conditions. The described method gives a good result, provided that the shear head height is correctly determined. On the other hand, it should be remembered that the analysis strictly covers the standard regulations, without undermining their credibility to be used for large dimensional supports. As noted in the first paragraphs of the paper, the considered standard methods may not give satisfactory results for large load fields.

The research program completed by the authors for selecting the shear head dimension allows for a proper reorganization of the punching shear capacity in slab–column connections. The authors hope that the described analytical method sparks a vital interest in the community of civil engineers and scientists to take into consideration the subject of the punching shear resistance of shear heads in building structures. The obtained results encourage the authors to continue the outlined research, also incorporating the extended theoretical and experimental investigations. The paper is intended to provide scientists, civil engineers, and designers with guidelines for the design process of the slab–column connections with the shear caps.

## Figures and Tables

**Table 1:** Values of the considered independent variables.

Variable	Lower Value	Basic Value	Upper Value
*ρ*_l,s_ (*A*_sl_)	0.25%	0.75%	1.25%
*l*_1_, *l*_2_	28 *h*_s_	33 *h*_s_	36 *h*_s_
*h*_s_	0.18 m	0.25 m	0.35 m
*c*_1_, *c*_2_	1.5 *h*_s_	2.0 *h*_s_	3.0 *h*_s_
*c*_nom_	0.05 *h*_s_	0.10 *h*_s_	0.15 *h*_s_

**Table 2:** Minimum values of the parameter $\alpha_{2}$, which satisfies the condition (2)—EC2.

α_1_	*c*_1_ = *c*_2_ = 1.5 *h*_s_	*c*_1_ = *c*_2_ = 2.0 *h*_s_	*c*_1_ = *c*_2_ = 3.0 *h*_s_
0.25	2.13	1.89	1.64
0.5	3.15	2.67	2.18
1	5.19	4.22	3.23
1.5	7.20	5.75	4.22
2	9.00	7.12	5.12

**Table 3:** Minimum values of the parameter $\alpha_{2}$, which satisfies the condition (2)—ACI318.

α_1_	*c*_1_ = *c*_2_ = 1.5 *h*_s_	*c*_1_ = *c*_2_ = 2.0 *h*_s_	*c*_1_ = *c*_2_ = 3.0 *h*_s_
0.25	1.70	1.63	1.89
0.5	2.85	2.85	2.85
1	6.05	5.39	4.58
1.5	9.20	7.60	6.00
2	-	-	7.10

**Table 4:** The shear heads dimensions determined in verification examples.

No.	*h*_s_ [cm]	*c*_s,1_ [cm]	*c*_s,2_ [cm]	*V*_Ed_ [kN]	*M*_Ed,1_ [kNm]	*M*_Ed,2_ [kNm]	EC2			ACI318		
							*h*_sh_ [cm]	*c*_sh,1_ [cm]	*c*_sh,2_ [cm]	*h*_sh_ [cm]	*c*_sh,1_ [cm]	*c*_sh,2_ [cm]
1	24	50	50	1185	34	54	45	185	185	35	135	134
2	33	40	40	2448	500	230	75	285	285	65	245	245
3	18	55	55	724	5	70	30	140	140	26	150	150
4	30	50	35	1188	260	21	45	180	125	38	80	55

## Appendix A. EC2 Verification Examples

The four verification examples are shown in Figure A1, Figure A2, Figure A3 and Figure A4. The notations used in Figure A1, Figure A2, Figure A3 and Figure A4 are collected and described in Table A1.

**Table A1:** Notations used in Appendix A.

	Notations
α1	relative height of the shear head (main parameter of the method)
α2	relative range of the shear head (main parameter of the method)
*h*_s_	slab height
*h*_tot_	total shear head height
*h*_sh_	height of the bold (from the underside of the shear head to underside of the slab)
*c*_nom,1_, *c*_nom,2_	the reinforcement cover in 1 and 2 directions
*d*_s,1_, *d*_s,2_	effective depth of slab in 1 and 2 directions
*d*_s_, *e*_ff_	effective depth of slab
*A*_s,1_, *A*_s,2_	reinforcement area in 1 and 2 directions
ρ_l,s,1_, ρ_l,s,2_	slab reinforcement ratio in 1 and 2 directions
ρ_l,s_	effective slab reinforcement ratio
*c*_s,1_, *c*_s,2_	column dimension in 1 and 2 directions
*c*_sh,1_, *c*_sh,2_	shear head dimension in 1 and 2 directions
*u*_0,s_	length of the control perimeter in the face of the support
*u*_1,s_	the length of the primary control perimeter
*k*	scale coefficient
f_ck_	characteristic compressive strength of concrete
γc	material safety factor for concrete
*v*_Rd,c1_	design shear strength of the unreinforced slab along the control perimeter under consideration
*v*_min_	minimum shear strength without reinforcement for puncture along the control perimeter under consideration
*v*_Rd,max_	design maximum shear strength without reinforcement for punching along the control perimeter under consideration
*v*_Ed,0_	design shear stress along the circumference at the face of the support under consideration
*v*_Ed,1_	design shear stress along the primary control perimeter of the support under consideration
*l*_1_, *l*_2_	slab span in 1 and 2 directions
*q*_Ed_	design total load evenly distributed over the slab generating the punching force
*V*_ed_	the punching force in the control perimeter under consideration
*ΔV*_Ed_	the part of the punching force generated from the load inside the field created by the control perimeter located at a distance of *d*_s.,eff_ from the face of the head
*M*_Ed,1_, *M*_Ed,2_	design unbalanced bending moment acting on the connection in 1 and 2 directions
*e*_1_, *e*_2_	eccentric of the punching force in the 1 and 2 directions
*b*_1,1_, *b*_2,1_	dimension of the primary control perimeter in the 1 and 2 directions
*b*_1,0_, *b*_2,0_	dimension of the control perimeter in the face of the support in 1 and 2 directions
*β*_1_, *β*_0_	coefficient increasing the tangential stress due to unbalanced moment acting on the connection in the primary control perimeter and at the face of the support

## Appendix B. ACI318 Verification Examples

The four verification examples are shown in Figure A5, Figure A6, Figure A7 and Figure A8. The notations used in Figure A5, Figure A6, Figure A7 and Figure A8 are collected and described in Table A2.

**Table A2:** Notations used in Appendix B.

	Notations
*α*_1_	relative height of the shear head (main parameter of the method)
*α*_2_	relative range of the shear head (main parameter of the method)
*h*_s_	slab height
*h*_tot_	total shear head height
*h*_sh_	height of the bold (from the underside of the shear head to underside of the slab)
*c*_nom,1_ *, c*_nom,2_	the reinforcement cover in 1 and 2 directions
*d*_s,1_ *, d*_s,2_	effective depth of slab in 1 and 2 directions
*d*_s_ *, e*_ff_	effective depth of slab
*A*_s1_ *, A*_s2_	reinforcement area in 1 and 2 directions
*c*_s,1_ *, c*_s,2_	column dimension in 1 and 2 directions
*c*_sh,1_ *, c*_sh,2_	shear head dimension in 1 and 2 directions
*b*_0,s_	length of the primary control perimeter for the column
*b*_0,sh_	length of the basic control perimeter for the head
*f*_ck_	characteristic compressive strength of concrete
*v*_c_	design shear strength of the unreinforced slab along the control perimeter under consideration
*v*_u_	design shear stress along the primary control perimeter of the support under consideration
*l*_1_ *, l*_2_	slab span in 1 and 2 directions
*q*_Ed_	design total load evenly distributed over the slab generating the punching force
*V*_ed_	the punching force in the control perimeter under consideration
*ΔV*_Ed_	the part of the punching force generated from the load inside the field created by the control perimeter
*M*_Ed,1_ *, M*_Ed,2_	design unbalanced bending moment acting on the connection in 1 and 2 directions
*b*_1,i_	the dimension of the primary control perimeter in a direction perpendicular to the axis in which the bending moment under consideration operates
*b*_2,i_	the dimension of the primary control perimeter in a direction parallel to the axis in which the bending moment under consideration operates
*γ*_v,i_	bending moment distribution coefficient for the direction under consideration
*c*_AB,i_	the distance from the center of gravity of the field bounded by the control perimeter to the limit of that perimeter in the direction under consideration
*J*_c,i_	the moment of inertia of the control section in relation to the center of gravity of the field bounded by that section
